# Umbilical Basal Cell Carcinoma With Mixed Nodular-Morpheaform Features and Stalk Invasion: A Case Report and Literature Review

**DOI:** 10.7759/cureus.88549

**Published:** 2025-07-22

**Authors:** Kazuyoshi Iijima, Kimika Ogihara, Airi Ohsawa, Megumi Hirabayashi, Yoshihiro Kuwano

**Affiliations:** 1 Department of Dermatology, Teikyo University Hospital, Mizonokuchi, Kawasaki, JPN

**Keywords:** basal cell carcinoma (bcc), morpheaform, nodular, rare coexistence, umbilicus

## Abstract

Basal cell carcinoma (BCC) is a common cutaneous malignancy; however, its occurrence in the umbilicus is exceptionally rare, with only 22 cases reported to date, including the present case. We describe a 79-year-old man presenting with a 3.0 × 1.9 cm grayish-brown umbilical nodule with central ulceration. Histopathological examination revealed mixed nodular and morpheaform features, with tumor extension into the umbilical stalk. Given the anatomical complexity of the umbilical region, preoperative imaging may be suboptimal for accurately assessing deep tumor extension due to interference from pre-existing scar tissue. Therefore, thorough histopathological evaluation, including intraoperative frozen section analysis, is essential to ensure complete excision and optimal surgical management.

## Introduction

Basal cell carcinoma (BCC) most commonly arises in sun-exposed areas, such as the head and neck [1]. Occurrence in non-sun-exposed regions is unusual, and involvement of the umbilicus is exceedingly rare. In addition, while BCC may display a range of histopathological subtypes, the presence of mixed subtypes within a single lesion is relatively uncommon and may be associated with more aggressive behavior [1]. Notably, morpheaform and infiltrative subtypes are considered particularly aggressive due to their propensity for deep tissue invasion and indistinct clinical margins [1].

Herein, we report a rare case of umbilical BCC exhibiting both nodular and morpheaform histology, with tumor infiltration into the umbilical stalk. We also provide a brief review of the literature on umbilical BCCs and discuss the implications for histological evaluation and surgical management in this anatomically complex region.

## Case presentation

**Figure 1:**
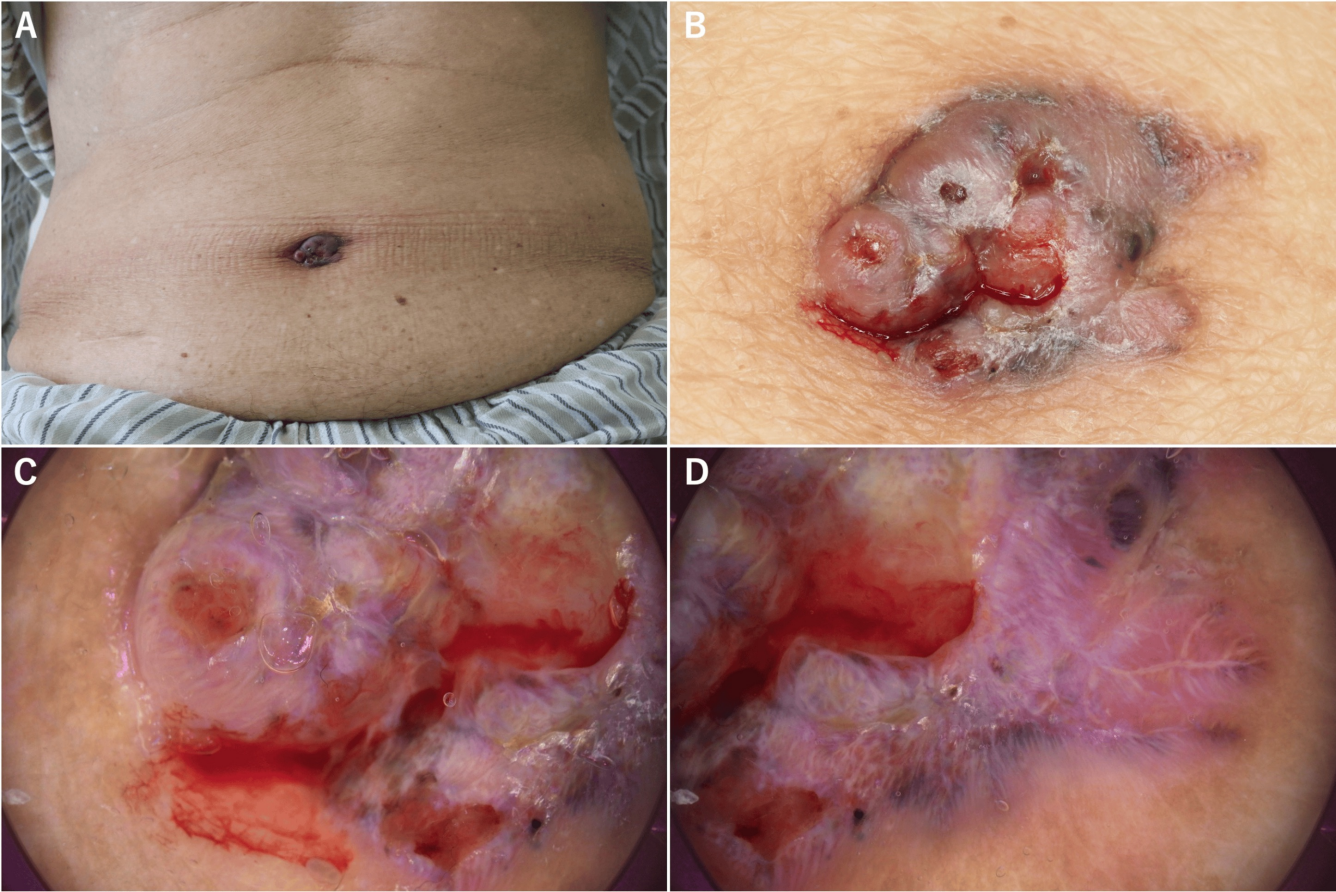
Distant and close-up views of an umbilical BCC A and B: The 3.0 × 1.9 cm lesion appears as a grayish-brown nodule with central ulceration. C and D: Dermoscopic findings of the lesion. The central area exhibits ulceration, with arborizing vessels, while the periphery shows leaf-like areas, large blue-gray ovoid nests, and multiple blue-gray globules. The surrounding surface is smooth and translucent. BCC: basal cell carcinoma

A 79-year-old man with Fitzpatrick skin type III presented with a 3.0 × 1.9 cm well-demarcated, grayish-brown nodule on the abdominal wall that had been gradually enlarging over the past six months. The lesion demonstrated central ulceration and was prone to bleeding (Figure 1A, 1B). His medical history included dementia, hypertension, and dyslipidemia, with no history of chronic umbilical inflammation, radiation therapy, or trauma to the umbilical region. Dermoscopy revealed central ulceration accompanied by arborizing vessels. The periphery exhibited leaf-like areas, large blue-gray ovoid nests, and multiple blue-gray globules. The surrounding surface was smooth with a translucent appearance (Figure 1C, 1D).

Based on these clinical findings, BCC was suspected. Several other brownish lesions were noted around the umbilicus. Dermoscopic findings of these lesions were consistent with seborrheic keratosis, with no evidence of malignancy. No additional suspicious findings or regional lymphadenopathy were present on examination or imaging. To exclude Sister Mary Joseph’s nodule or other primary umbilical tumors, a punch biopsy was performed. Histopathological examination revealed nests of basaloid cells continuous with the epidermis, with melanin deposition within the nests, consistent with nodular BCC.

**Figure 2:**
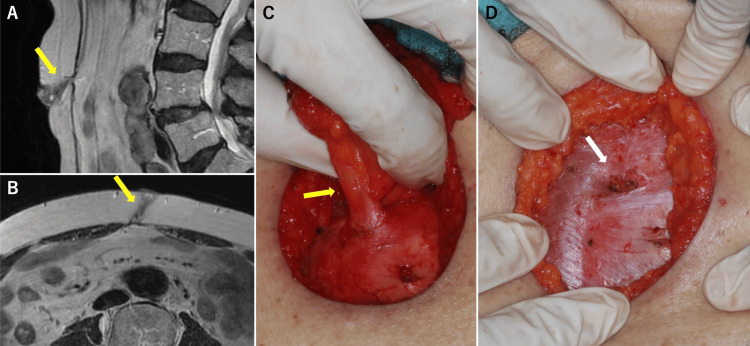
Umbilical BCC: MRI findings and intraoperative photographs A (sagittal view) and B (axial view): A 30 mm nodule located in the umbilicus shows high signal intensity on T2-weighted imaging. The yellow arrows indicate the umbilical stalk. C: Tumor excision was performed at the level of the rectus sheath while maintaining continuity with the umbilical stalk (yellow arrow). D: After excision of the umbilical stalk en bloc at the preperitoneal fat level, small defects were observed in the rectus sheath (white arrow). BCC: basal cell carcinoma, MRI: magnetic resonance imaging

Magnetic resonance imaging (MRI) revealed a 30 mm nodule in the umbilical region with high signal intensity on T2-weighted imaging (Figure 2A, 2B). Although no definitive invasion of the muscle layer or peritoneum was observed, the tumor overlapped with the low-signal area of the umbilical stalk, complicating an accurate assessment of invasion depth.

The tumor was surgically excised under general anesthesia with a 10 mm circumferential margin. Excision was performed at the level of the rectus sheath, with en bloc resection of the umbilical stalk down to the preperitoneal fat, sparing the peritoneum (Figure 2C, 2D). Intraoperative frozen section analysis confirmed negative deep margins. A defect in the rectus sheath was repaired with 3-0 Vicryl, and soft tissue was closed in layers using 3-0 polydioxanone (PDS) Ⅱ. The wound was closed with simple sutures, and a Penrose drain was placed. The patient opted against umbilical reconstruction.

**Figure 3:**
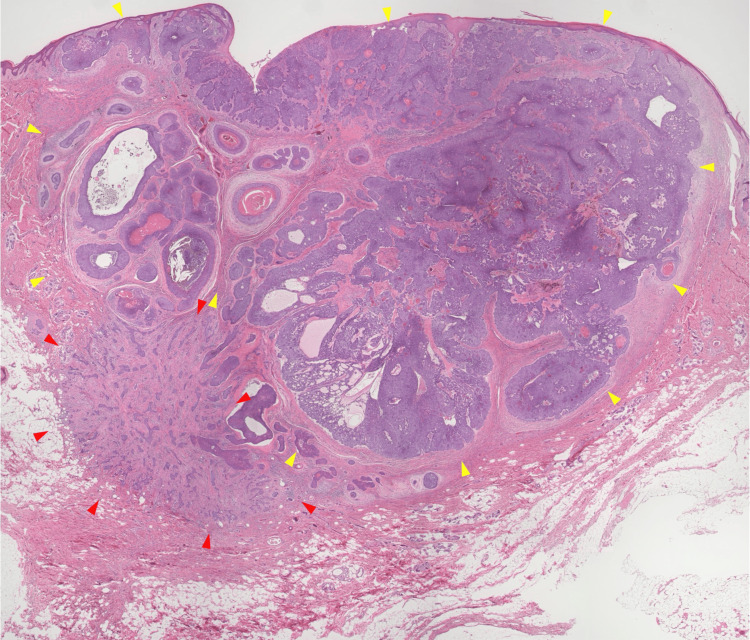
Umbilical BCC: histopathological overview Nodular-type basaloid tumor cells are observed in the dermis in continuity with the epidermal basal layer (yellow arrowheads). At the dermo-subcutaneous junction, small nests of tumor cells embedded in dense fibrous stroma, indicative of a morpheaform component, are also observed (red arrowheads) (H&E, ×200). BCC: basal cell carcinoma, H&E: hematoxylin and eosin

**Figure 4:**
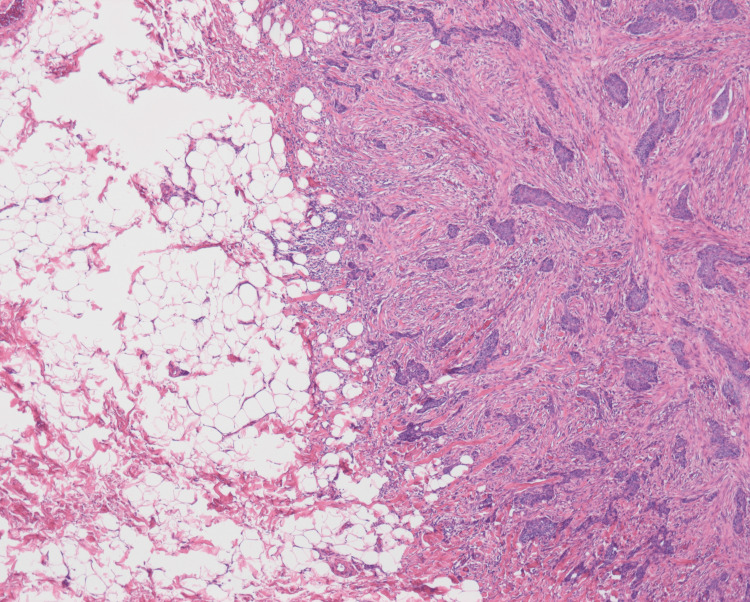
Umbilical BCC: morpheaform component (high-power view) Higher magnification of the dermo-subcutaneous junction is shown in Figure 4. Small basaloid tumor nests are embedded in dense fibrous stroma, consistent with a morpheaform component, predominantly located in the dermis with focal infiltration into the subcutis (H&E, ×400). BCC: basal cell carcinoma, H&E: hematoxylin and eosin

Histopathological analysis revealed a predominant nodular component in the dermis, characterized by basaloid tumor nests extending from the epidermal basal layer with peripheral palisading and stromal clefting (Figure 3). A morpheaform component was also present, represented by small nests of basaloid cells embedded in dense fibrous stroma at the dermo-subcutaneous junction, predominantly within the dermis with focal extension into the subcutis (Figures 3, 4).

**Figure 5:**
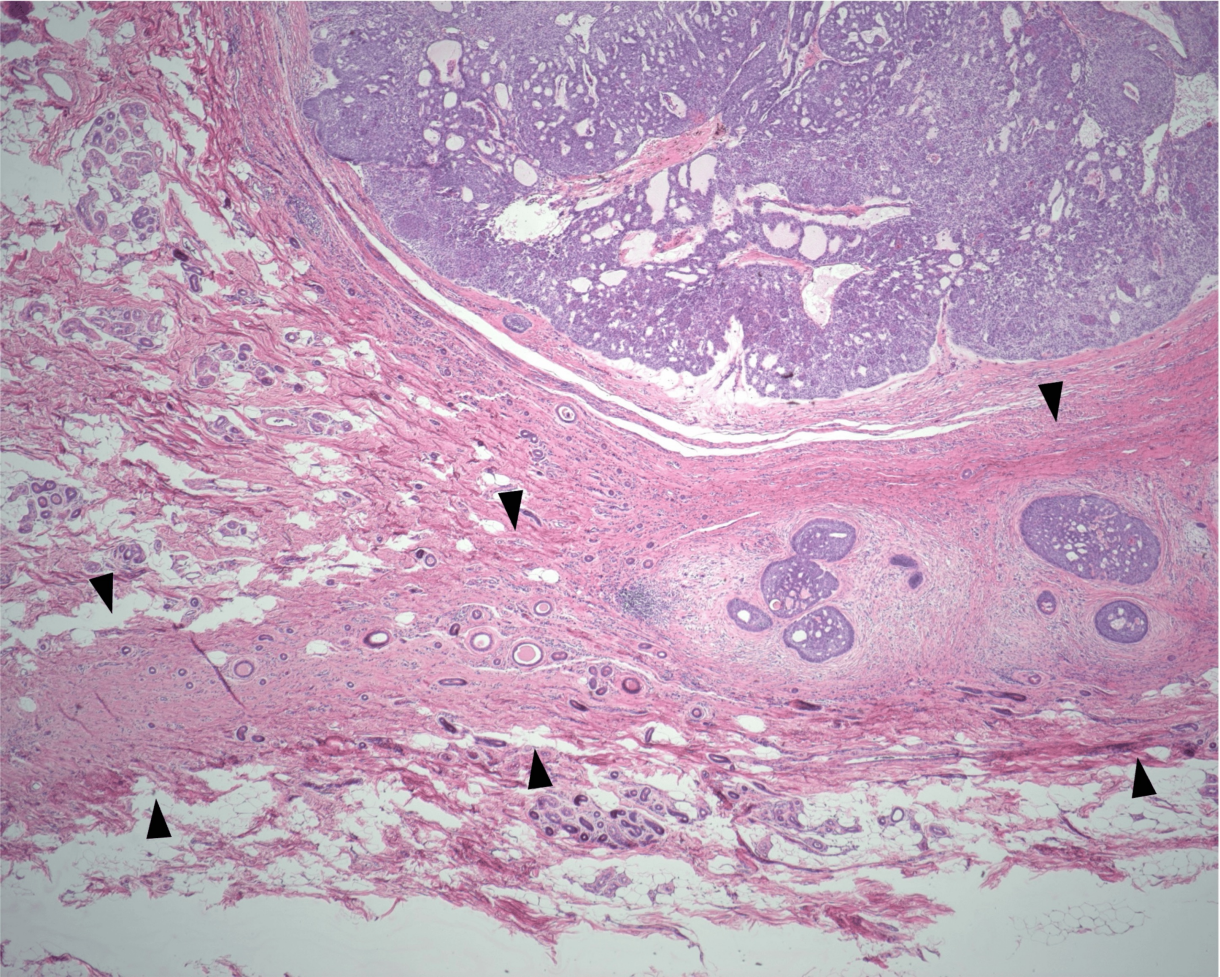
Umbilical BCC: nodular component in the umbilical stalk The umbilical stalk (arrowheads) contains nests of basaloid tumor cells exhibiting peripheral palisading, consistent with a nodular growth pattern (H&E, ×200). BCC: basal cell carcinoma, H&E: hematoxylin and eosin

Basaloid tumor nests with nodular architecture were additionally identified within the umbilical stalk (Figure 5). No evidence of perineural invasion was identified on histopathological examination. Furthermore, no significant infiltrates, such as mast cells, were observed.

## Discussion

BCC is the most common cutaneous malignancy, predominantly associated with chronic ultraviolet (UV) exposure. Approximately 80%-85% of cases occur in sun-exposed areas, particularly the head and neck, while less than 2% arise in sun-protected regions, including the axillae, abdomen, and groin [2]. Among these, umbilical involvement remains exceptionally rare. Additional risk factors include chronic inflammation, prior trauma or surgery, and ionizing radiation.

Primary malignancies of the umbilicus are uncommon, whereas metastatic tumors, known as Sister Mary Joseph’s nodules, account for approximately 80% of umbilical malignancies [3]. Primary neoplasms of the umbilicus include urachal adenocarcinoma, myosarcoma, malignant melanoma (MM), extramammary Paget’s disease, squamous cell carcinoma, and, rarely, BCC. A review of 112 umbilical tumors identified only eight primary malignancies (7.1%), of which MM was the most frequent (four cases), followed by BCC (two cases (1.8%)) [3].

A total of 21 reported cases of umbilical basal cell carcinoma were identified through a PubMed search (1965-June 2025) using the terms “umbilical basal cell carcinoma” and “basal cell carcinoma of the umbilicus.” Relevant cases cited within the retrieved articles were also reviewed and included as appropriate. Including the present case, a total of 22 cases have been summarized (Table 1).

**Table 1:** Reported cases of umbilical basal cell carcinoma Cases were identified through a PubMed search (1965-June 2025) using the terms “umbilical basal cell carcinoma” and “basal cell carcinoma of the umbilicus,” along with relevant articles cited therein. The current case is included. Bold text in the “Histopathology” column indicates cases that demonstrated mixed histological types (e.g., nodular + morpheaform). Bold text in the “Infiltration level” column indicates cases that involved umbilical stalk infiltration. NA: not available, US: ultrasound, CT: computed tomography, MRI: magnetic resonance imaging, MMS: Mohs micrographic surgery, FEP: fibroepithelioma of Pinkus, CO₂: ultrapulse CO₂ laser

Author	Age and sex	Size (cm)	Morphology	Histopathology	Image	Infiltration level	Treatment
Steck and Helwig [3]	76, female	NA	NA	NA	NA	NA	Surgery
Steck and Helwig [3]	52, male	NA	Pigmented multicentric	NA	NA	NA	Surgery
Schneider and Young [4]	71, female	NA	NA	NA	NA	NA	Surgery
Krunic et al. [5]	48, male	6.5 × 4.5	Plaque	Nodular + superficial	NA	NA	Surgery, CO_2_
Walker et al. [6]	27, female	0.4 × 0.4	Nevus	Nodular	NA	NA	Surgery
Etter and Cook [7]	43, female	2.7 × 1.5	Red scary plaque	Superficial	NA	NA	MMS
Durrani et al. [8]	54, female	2.5	NA	Nodular	NA	NA	Surgery
Chuang et al. [9]	80, female	2.0 × 1.8	Erythematous, pigmented plaque	Nodular	NA	Surface of transversalis fascia with umbilical stalk involvement	MMS, radiation
Ramirez et al. [10]	21, male	NA	Nonpigmented papule	Nodular	NA	NA	Surgery
Lin et al. [11]	67, female	NA	Erythematous, dark brownish to bluish mass	FEP + nodulocystic	NA	NA	Surgery
Ramos-e-Silva et al. [12]	75, female	NA	NA	NA	NA	NA	Surgery
Nakamura et al. [13]	94, female	2.0 × 2.0	Dark bluish mass	Nodular	CT	Bottom of the umbilicus	Surgery
Nakamura et al. [13]	54, male	4.0 × 3.0	Reddish-black mass	Nodular	CT	Middle layer of the abdominal wall	Surgery
Nakamura et al. [13]	68, female	1.8 × 1.5	Dark-bluish mass	Nodular	NA	Bottom of the umbilicus	Surgery
Narala and Cohen [14]	50, female	3.0 × 1.5	Pigmented plaque	Nodular	NA	NA	Surgery
Inskip et al. [15]	83, male	0.1 × 0.1	Soft polypoid	FEP	NA	NA	NA
Orduz Robledo et al. [16]	54, male	14 × 10	Ulcerated wound with infiltrated nodular borders	Morpheaform	US, CT, MRI	Abdominal fascia	Vismodegib, surgery
Shen et al. [17]	78, female	3.0 × 2.0	Ulcerated wound	Nodular	CT	Subcutaneous tissue with umbilical stalk involvement	Surgery
Takada et al. [18]	72, male	1.5 × 0.7	Pigmented nodule	Superficial	NA	NA	Surgery
Kurosaki et al. [19]	61, female	2.0 × 0.3	Black plaque	Superficial	CT	NA	Surgery
Kurosaki et al. [19]	80, female	0.7 × 1.5	Dark brownish plaque	Superficial	NA	NA	Surgery
Current case	79, male	3.0 × 1.9	Grayish-brown nodule with ulceration	Nodular + morpheaform	US, CT, MRI	Subcutaneous tissue with umbilical stalk involvement	Surgery

The median patient age was 67.5 years (range: 21-94), with a notable difference between male patients (median: 54 years) and female patients (median: 69.5 years). Due to variability in the level of detail across published reports, only cases with clearly documented clinical or histopathological features were included in the analyses. In cases with mixed histological subtypes, each component was counted separately, which may have resulted in overlap. Clinically, umbilical BCCs most commonly present as plaques (eight of 18 cases, 44%) [5,7,9,14,16,17,19], followed by nodules, masses, or papules (seven of 18 cases, 39%) [10,11,13,18].

Histologically, the nodular subtype is the most prevalent (12 of 18 cases, 67%), followed by superficial (five of 18 cases, 28%), morpheaform (two of 18 cases, 11%), and fibroepithelial variants (two of 18 cases, 11%). Mixed histological patterns were observed in three of 18 cases (17%).

The superficial subtype predominantly presented as plaques (four of five cases, 80%), whereas the nodular subtype typically manifested as papules or nodules (six of 12 cases, 50%). Morpheaform BCC, which exhibits a greater propensity for deep infiltration, often appeared as indurated plaques and occasionally ulcerated. These findings align with the general morphological patterns of BCC described by Marzuka et al., underscoring their relevance to umbilical lesions [20].

Our case is the first documented instance of umbilical BCC with a mixed nodular and morpheaform histological pattern. While morpheaform and infiltrative subtypes are generally considered more aggressive, nodular BCC is typically less invasive. However, our case demonstrated basaloid tumor cell nests with peripheral palisading within the umbilical stalk, confirming nodular-type infiltration. The morpheaform nests were located within the dermis, with evidence of infiltration into the subcutaneous fat, but with no evidence of deeper invasion into the stalk. Among previously reported umbilical BCC cases, two demonstrated invasions to the stalk [9,17]. Notably, both cases were of the nodular subtype, suggesting that even histologically non-aggressive variants may invade the stalk when in close proximity.

The umbilicus, a critical anatomical landmark, is a physiological scar from the umbilical cord. Several embryological remnants contribute to its structure, including the median umbilical ligament (obliterated urachus), medial umbilical ligaments (obliterated umbilical arteries), and falciform ligament (remnant of the umbilical vein). At its core, the umbilical stalk passes through the umbilical ring, penetrating the linea alba, which is formed by the fusion of the right and left rectus sheaths. These layers are composed of the aponeuroses of the external oblique, internal oblique, and transversus abdominis muscles. Deep to these structures lie the transversalis fascia and preperitoneal fat, followed by the peritoneum as the deepest layer. Given the complex anatomical arrangement and proximity of these structures, the umbilicus may serve as a potential conduit for deeper tumor invasion, theoretically allowing extension into the peritoneum or abdominal cavity via the umbilical stalk. While peritoneal involvement has not been documented in any reported cases, two cases exhibited stalk infiltration, with one extending to the transversalis fascia [9]. These findings underscore the need for meticulous histopathological assessment to evaluate the risk of deeper extension.

For large umbilical BCCs or those with a high risk of deep extension, preoperative imaging with MRI or CT is often used to assess deep margins. However, in the umbilical region, scar tissue often interferes with imaging, limiting its accuracy. Intraoperative frozen section analysis is therefore critical to ensuring complete excision and minimizing recurrence risk. In our case, intraoperative pathology confirmed negative deep margins, eliminating the need for further excision. Given the umbilicus’ proximity to deeper structures, positive margins would have necessitated additional resection, potentially extending to the peritoneum.

## Conclusions

This case is the first reported instance of umbilical BCC with mixed nodular and morpheaform histology. Given the anatomical complexity of the umbilical region, thorough histopathological evaluation is essential to assess tumor extent and guide surgical management. Precise margin control, particularly in cases involving the umbilical stalk, is important due to the anatomical proximity to the peritoneum, although peritoneal invasion has not been observed in reported cases. Collaboration between pathologists and surgeons, along with intraoperative frozen section analysis, plays a key role in optimizing clinical outcomes in these rare cases.
